# Characterization of Subcellular Organelles in Cortical Perisynaptic Astrocytes

**DOI:** 10.3389/fncel.2020.573944

**Published:** 2021-01-28

**Authors:** Amina Aboufares El Alaoui, Molly Jackson, Mara Fabri, Luisa de Vivo, Michele Bellesi

**Affiliations:** ^1^Department of Experimental and Clinical Medicine, Marche Polytechnic University, Ancona, Italy; ^2^School of Physiology, Pharmacology, and Neuroscience, University of Bristol, Bristol, United Kingdom

**Keywords:** astrocyte, synapse, perisynaptic astrocytic processes, electron microscopy, mouse

## Abstract

Perisynaptic astrocytic processes (PAPs) carry out several different functions, from metabolite clearing to control of neuronal excitability and synaptic plasticity. All these functions are likely orchestrated by complex cellular machinery that resides within the PAPs and relies on a fine interplay between multiple subcellular components. However, traditional transmission electron microscopy (EM) studies have found that PAPs are remarkably poor of intracellular organelles, failing to explain how such a variety of PAP functions are achieved in the absence of a proportional complex network of intracellular structures. Here, we use serial block-face scanning EM to reconstruct and describe in three dimensions PAPs and their intracellular organelles in two different mouse cortical regions. We described five distinct organelles, which included empty and full endosomes, phagosomes, mitochondria, and endoplasmic reticulum (ER) cisternae, distributed within three PAPs categories (branches, branchlets, and leaflets). The majority of PAPs belonged to the leaflets category (~60%), with branchlets representing a minority (~37%). Branches were rarely in contact with synapses (<3%). Branches had a higher density of mitochondria and ER cisternae than branchlets and leaflets. Also, branches and branchlets displayed organelles more frequently than leaflets. Endosomes and phagosomes, which accounted for more than 60% of all the organelles detected, were often associated with the same PAP. Likewise, mitochondria and ER cisternae, representing ~40% of all organelles were usually associated. No differences were noted between the organelle distribution of the somatosensory and the anterior cingulate cortex. Finally, the organelle distribution in PAPs did not largely depend on the presence of a spine apparatus or a pre-synaptic mitochondrion in the synapse that PAPs were enwrapping, with some exceptions regarding the presence of phagosomes and ER cisternae, which were slightly more represented around synapses lacking a spine apparatus and a presynaptic mitochondrion, respectively. Thus, PAPs contain several subcellular organelles that could underlie the diverse astrocytic functions carried out at central synapses.

## Introduction

Over the last decades, astrocytes have been assigned an increasing number of functions, from neurovascular coupling and metabolite clearing, to control of neuronal excitability and synaptic plasticity (Nuriya and Hirase, 2016; Papouin et al., 2017; Mestre et al., 2020). Recent evidence suggests that these functions may be specialized depending on astrocyte localization within the central nervous system. For example, it has been demonstrated that astrocytes show molecular and physiological heterogeneity within different layers of the cerebral cortex and across different brain regions (Miller, 2018; Xin and Bonci, 2018).

Astrocytes display a spongiform shape and are equipped with processes that infiltrate the neural tissue reaching out to synapses that tend to encapsulate (Reichenbach et al., 2010). Such processes can be morpho-functionally classified into branches, branchlets, leaflets, and endfeet, depending on size and location. Branches are the largest processes departing from the soma with a diameter on the micrometer scale. Branchlets are higher-order processes emanating from branches with a size in the order of the submicrometer scale. Leaflets and endfeet are the finest processes contacting the synapses and blood vessels, respectively (Patrushev et al., 2013; Khakh and Sofroniew, 2015). It has been estimated that up to 75% of cortical and 65% of hippocampal axo-spinous synapses are contacted by astrocytic processes (Witcher et al., 2007; Lushnikova et al., 2009; Bernardinelli et al., 2014). This close anatomical relationship with synapses is necessary to allow astrocytes to sense and modulate the synaptic environment (Reichenbach et al., 2010; Bernardinelli et al., 2014; Heller and Rusakov, 2015). When surrounding the synapses, the astrocytic processes are commonly named perisynaptic astrocytic processes (PAPs). This term usually refers to leaflets, the structures that more frequently are in close contact with synapses. However, it is worth noting that synapses can be contacted also by branchlets and branches.

PAPs plasma membrane is enriched in transporters that are essential to reuptake neurotransmitters released in the synaptic cleft and control neurotransmitter spillover to extrasynaptic sites and neighbor synapses (Kullmann, 2000; Melone et al., 2009; Bellesi and Conti, 2010; Murphy-Royal et al., 2017). Also, PAPs regulate the extracellular ion concentration and water homeostasis and provide energy substrates (e.g., lactate) to synapses (Papadopoulos and Verkman, 2013; Magistretti and Allaman, 2018; Nakada and Kwee, 2019). PAPs participate in the formation of the tripartite synapse, a structural and functional interrelationship between neuronal and astrocytic elements that leads the astrocyte to sense synaptic activity and react through the release of synaptic active molecules (e.g., gliotransmitters; Halassa et al., 2007; Perea et al., 2009; Savtchouk and Volterra, 2018). PAPs are far from being static structures. On the contrary, they can move and remodel by shrinking or expanding very quickly in a way that is dependent on synaptic activity and animal behavior (Genoud et al., 2006; Bernardinelli et al., 2014; Bellesi et al., 2015). Furthermore, recent studies demonstrated that PAPs possess phagocytic skills and are capable of engulfing portions of synapses, thus playing an important role in sculpting neuronal circuits during development and in response to stressful conditions like the lack of sleep (Chung et al., 2013; Bellesi et al., 2017).

All these functions are likely orchestrated by complex cellular machinery that resides within the PAPs and relies on a fine interplay between multiple subcellular components. Historically, PAPs have been described as structures lacking many organelles, such as mitochondria and endoplasmic reticulum (ER), which are found more frequently in large astrocytic processes such as branchlets or branches (Peters, 1991; Reichenbach et al., 2010; Patrushev et al., 2013; Bernardinelli et al., 2014; Gavrilov et al., 2018). However, other studies performed in *in vitro* and in *ex-vivo* preparations have challenged this view by showing the presence of small mitochondria and other organelles such as vesicles, endosomes, or phagosomes in PAPs (Milanese et al., 2009; Sahlender et al., 2014; Volterra et al., 2014; Bellesi et al., 2015, 2017; Cervetto et al., 2015; Derouiche et al., 2015). Thus, a more detailed morphological characterization that better fits with the plethora of functions that PAPs normally carry out is required.

PAPs are usually too small to be studied using light microscopy techniques, although novel imaging methods using genetically expressed tags have improved their detection and analysis (Bindocci et al., 2017). Electron microscopy (EM) remains the gold standard to study PAPs, as it offers an adequate spatial resolution that allows a further dissection of PAP subcellular components. Since the preparation and acquisition of samples for serial section transmission EM are time-consuming, most ultrastructural studies are limited to the analysis of small portions of tissue and organelles. By contrast, new volume EM techniques, such as serial block-face EM (SBF-SEM), permit automatic cutting and imaging of large portions of brain tissue (e.g., 1 cubic millimeter) thus allowing the identification and quantification of thousands of structures in three-dimensions (Hughes et al., 2014; Bellesi et al., 2015; de Vivo et al., 2017). Volumetric reconstruction of cellular structures facilitates the identification process and provides a better and more effective snapshot of PAP morphology relative to two-dimensional imaging techniques (Bellesi et al., 2015).

Here, we employed SBF-SEM to study the subcellular composition of PAPs surrounding axo-spinous synapses of layer II in two cortical regions, the somatosensory (SS) and the anterior cingulate (AC) cortex, and to identify undescribed features of PAPs, which may help to explain their complex behavior.

## Materials and Methods

### Ultrastructural Studies

C57BL6J male mice (4 weeks of age) were kept under a 12-h dark-light cycle (8 AM–8 PM) and permitted food and water *ad libitum*. Between 2 and 4 PM, mice were perfused intracardially under deep anesthesia with a solution of 0.05 M phosphate-buffered saline followed by 2.5% glutaraldehyde and 4% paraformaldehyde dissolved in 0.1 M sodium cacodylate buffer (41°C and pH 7.4). Brains were removed and kept in the same fixative overnight at 4°C. Brain slices were cut on a vibratome and kept in a cryoprotectant solution until the day of processing. Sections were rinsed 3 × 10 min each in cacodylate buffer and incubated for 1 h on ice with a solution of 1.5% potassium ferrocyanide/2% osmium tetroxide. After three rinses in double distilled water, they were exposed to a solution of 1% thiocarbohydrazide for 20 min at room temperature. Sections were washed with distilled water and placed in 2% osmium tetroxide for 30 min, washed again, and incubated overnight with 1% uranyl acetate at 4°C. The following day, the tissue was stained with a solution of lead aspartate, dehydrated, and embedded with Durcupan resin and ACLAR film. Small squares of tissue from the AC and SS cortex (layer II–III) were glued on the tip of a metal pin and coated with silver paint to minimize specimen charging during imaging.

### Image Acquisition and Analysis

Images were obtained using serial block-face electron microscope—ΣIGMA^TM^ VP field emission scanning electron microscope (Carl Zeiss NTS Limited) equipped with 3View^®^ technology (Gatan Inc.), and a backscattered electron detector. Images were acquired using an aperture of 30 μm, high vacuum, an acceleration voltage of 1.2 kV, with image resolution (*xy* plane) between 4 and 6 nm. Serial images were obtained by scanning the face of an unsliced block of tissue placed inside the microscope, then cutting off ultrathin slices using an automated microtome within the instrument (50 nm thickness). The newly exposed surface of a sliced block was rescanned until a stack of images was obtained. The series of images were processed and analyzed using FIJI. TrakEM2, a FIJI plug-in, was used for the segmentation of neuropil elements. Axo-spinous asymmetric (putative excitatory) synapses were identified in the neuropil. For each synapse, we defined the axon–interface (ASI) as the interfacing surface between the spine and the axonal bouton, and a cuboid region of interest (ROI) was drawn around the synapse. ROI included the axon terminal, the post-synaptic element, and the peri-synaptic astrocyte (PAP) when present. PAPs were recognized based on their distinctive shapes, interdigitating among neuronal profiles and often contacting parts of the synapse, and on the presence of glycogen granules. Other elements of the neuropil such as cell bodies, blood vessels, or large dendrites were not included in the ROI. For each ROI, two blinded scorers segmented the spine head, the ASI, the PAPs, and identified all the organelles included in the PAP. PAPs were classified as branches (process diameter >800 nm), branchlets (process diameter: 250–800 nm), and leaflets (process diameter: <250 nm). In these structures, five different types of organelles were recognizable: (1) empty endosomes (EE), described as vesicular structures with a diameter of 80–150 nm often showing a clear endoplasm with no inclusions; (2) full endosomes (FE) described as endosomes containing smaller vesicles or amorphic material; (3) phagosomes (PH) described by the presence of a portion of axon, spine head, or dendrite being invaginated by the surrounding PAP, with a clear continuity between the part being enclosed by the PAP (phagosome) and the neuronal structure; (4) Mitochondria (MT); and (5) ER cisternae (ER) described as long membranous structures. We also estimated the number of synapses contacted by PAP at the post-synaptic site (spine + synapses) or cleft level (ASI+ synapses) and the extent of synaptic coverage quantified as the interfacing apposed surface between the PAP and the postsynaptic element. To estimate whether organelles are isolated or tend to form a group, we performed a cluster analysis by correlating the occurrence frequency within the same PAP of two or more organelles.

### Statistical Analysis

Parametric statistics were used in this study. We used paired *t*-test, repeated measure ANOVA, or mixed models when values were missing to evaluate the difference between cortical regions and Pearson’s correlations to assess relations between variables. Alpha was set to 0.05 but opportunely corrected in case of multiple comparisons.

## Results

### PAPs Surrounding Axo-Spinous Synapses

To characterize the subcellular composition of PAPs in relation to axo-spinous synapses, we considered two different brain regions, SS and the AC cortex of adolescent mice (*n* = 4). We hypothesized that since these two cortical regions are involved in different brain functions and belong to a different hierarchical order, they would also have a heterogenous organelle composition within PAPs.

**Figure 1:**
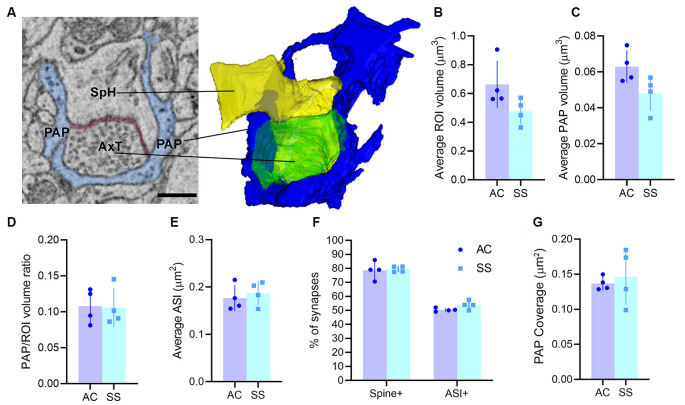
**(A)** Example of region of interest (ROI) showing an axo-spinous synapse with perisynaptic astrocytic process (PAP; blue), axon terminal (AxT), spine head (SpH), axon-spine interface (ASI, red), and relative 3D reconstruction. Scale bar: 400 nm. **(B,C)** Average ROI and PAP volumes, respectively. **(D)** Ratio between PAP and ROI volumes. **(E)** Average ASI size. **(F)** Percentage of synapses contacting the spine (spine+) and the synaptic cleft (ASI+). **(G)** Synaptic coverage as measured by the size of the interfacing surface between the PAP and the spine head. In panels **(B–G)** AC and SS refer to anterior cingulate and somatosensory cortex (paired *t*-test, *n* = 4).

By following astrocytic profiles in three dimensions, PAPs surrounding axo-spinous synapses were easily identified: they often displayed sharp angles, contained glycogen granules, and interdigitated between neuronal structures. For each synapse, we reconstructed the pre-and post-synaptic elements and the PAPs within a three-dimensional ROI. In the two cortical regions, we sampled a comparable volume of neuropil (ROI volume, AC: 0.66 ± 0.16 μm^3^; SS: 0.48 ± 0.09 μm^3^; *p* = 0.09), PAP volume (AC: 0.06 ± 0.01 μm^3^; SS: 0.05 ± 0.01 μm^3^; *p* = 0.07; ratio between PAP and ROI volume in AC: 0.1 ± 0.01, in SS: 0.1 ± 0.02; *p* = 0.7; Figures 1A–D), and synapses of similar size, as shown by the distribution of ASI measures (AC: 0.18 ± 0.03 μm^2^; SS: 0.19 ± 0.03 μm^2^; *p* = 0.6; Figure 1E). We found that the fraction of synapses contacted by a PAP at the post-synaptic site was 78.6 ± 6.3% for AC and 79.4 ± 2.1% for SS (*p* = 0.8), while the fraction of synapses contacted by PAP at the synaptic cleft was 50.39 ± 1.27% for AC and 53.79 ± 3.09% for SS (*p* = 0.1; Figure 1F). Synapses not contacted by PAP represented 22.4 ± 6.3% for AC and 21.6 ± 2.1% for SS (*p* = 0.8; not shown). Finally, we found that the extent of PAP coverage around the post-synaptic element was comparable between the two cortical regions (AC: 0.14 ± 0.01 μm^2^; SS: 0.15 ± 0.04 μm^2^; *p* = 0.65; Figure 1G).

### PAPs Contain Multiple Subcellular Organelles

**Figure 2:**
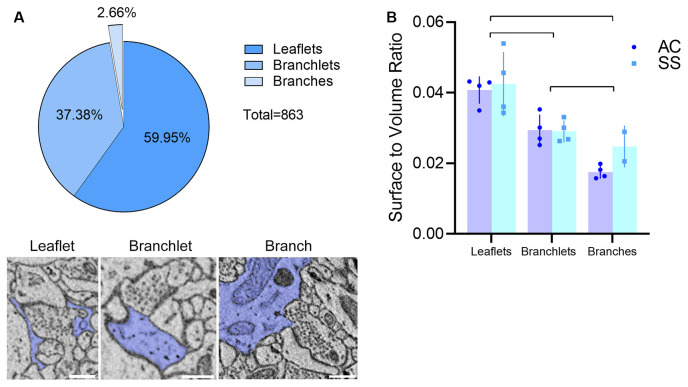
**(A)** Top: PAP composition in our dataset (*n* = 863, four animals). Bottom: examples of the leaflet, branchlet, and branch depicted in blue and surrounding an axo-spinous synapse. The scale bar is 350 nm for all panels. **(B)** Average surface-to-volume ratio values in AC and SS (mixed-effects analysis, *n* = 4). Lines indicate significant pairwise comparisons (*p* < 0.05, Bonferroni corrected).

**Figure 3:**
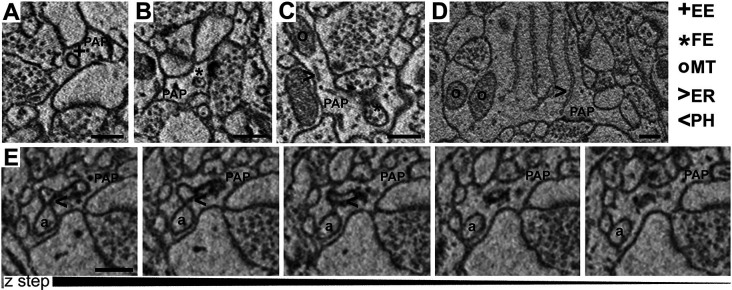
**(A–D)** Examples of empty endosomes (EE), full endosomes (FE), mitochondria (MT), ER-cisternae (ER). **(E)** Axon (a) being engulfed by a PAP caught in several consecutive *z*-steps showing the formation of the phagosome (PH). Scale bar is 300 nm for all panels.

**Figure 4:**
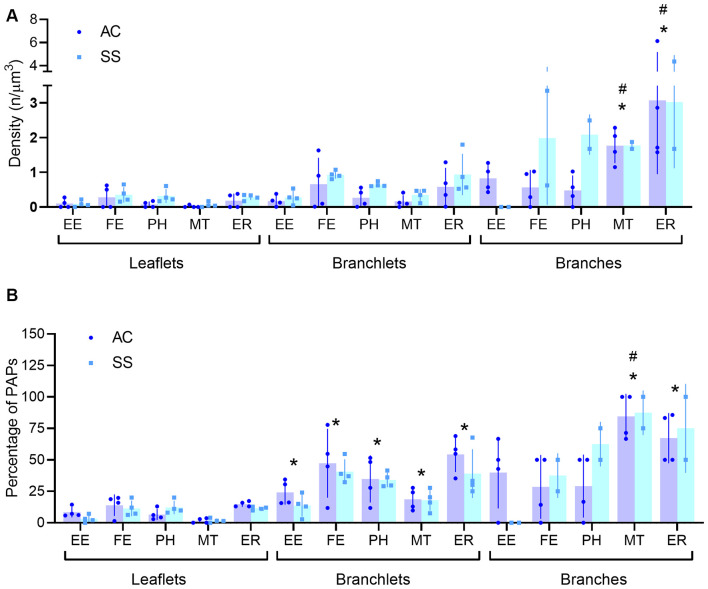
**(A,B)** Quantification of PAP subcellular organelle distribution **(A)** and percentage of perisynaptic astrocytic processes (PAPS) displaying at least one organelle **(B)**. All data are segregated for leaflets, branchlets, and branches (*n* = 4). Empty endosomes (EE), full endosomes (FE), phagosome (PH), mitochondria (MT), and ER cisternae (ER) for all panels. *Indicates significant pairwise comparisons (mixed-effects analysis, *p* < 0.05, Bonferroni corrected) relative to leaflets, whereas, the ^#^indicates significant pairwise comparisons (mixed-effects analysis, *p* < 0.05, Bonferroni corrected) relative to branchlets.

Visual inspection of 863 manually segmented PAPs surrounding axo-spinous synapses of the SS and AC cortex identified 23 branches (2.66%), 323 branchlets (37.38%), and 517 leaflets (59.95%; Figure 2A). As expected, branches, branchlets, and leaflets displayed different values of surface to volume ratio, with leaflets having a more convoluted and less rounded shape than branchlets and branches (Figure 2B). We detected five classes of subcellular organelles in PAPs: these classes included: (1) EE; (2) FE; (3) PH; (4) MT; and (5) ER (Figures 3A–E). We applied a mixed-effect model (REML) with cortical region and type of organelle as fixed factors to assess the organelle distributions in branches, branchlets, and leaflets. While cortical region did not influence the distribution of these organelles within branches, branchlets, and leaflets (*p* = 0.13), we found that the density of ER and MT was significantly higher in branches relative to branchlets and leaflets (*p* < 0.0001; Figure 4A). No significant differences were found between leaflets and branchlets.

When evaluating the fraction of branches, branchlets, and leaflets containing at least one of the mentioned organelles, we found that leaflets had organelles in 8.54 ± 5.02% of the cases, branchlets in 32.49 ± 13.5%, and branches in 47.22 ± 21.8%. Leaflets contained organelles less frequently than branchlets, for all classes of organelles [REML with cortical region and type of organelle as fixed factors, effect found for type of organelles *p* < 0.0001;* post hoc* tests for EE (*p* = 0.046), FE (*p* = 0.022), PH (*p* = 0.02), MT (*p* = 0.022), ER (*p* = 0.016)], whereas the difference was significant only for MT (*p* = 0.01) and ER (*p* = 0.021) when leaflets were compared to branches. Among all organelles, mitochondria were the least present in leaflets. When comparing branchlets and branches, we found that the fraction of branches containing MT was higher than the branchlets’ one (*p* = 0.002; Table 1, Figure 4B).

**Table 1:** Percentage of PAPs containing at least one organelle (mean ± std, *n* = 4).

			*AC*					*SS*		
	EE	FE	PH	MT	ER	EE	FE	PH	MT	ER
Leaflets	8.4 ± 4.1	14.0 ± 8.5	6.6 ± 4.5	1.7 ± 1.9	14.8 ± 2.1	3.1 ± 3.0	11.4 ± 6.2	12.2 ± 5.3	1.8 ± 1.7	11.4 ± 1.3
Branchlets	24.2 ± 9.7	47.3 ± 27.4	34.9 ± 18.7	18.7 ± 8.7	54.4 ± 14.1	13.7 ± 8.7	40.7 ± 9.7	34.1 ± 5.9	18.0 ± 8.4	39.1 ± 19.4
Branches	39.9 ± 28.4	28.6 ± 25.4	29.2 ± 20.8	72.0 ± 20.8	61.0 ± 29.0	17.1 ± 16.4	32.3 ± 14.3	42.2 ± 27.1	67.0 ± 40.5	60.0 ± 36.5

**Figure 5:**
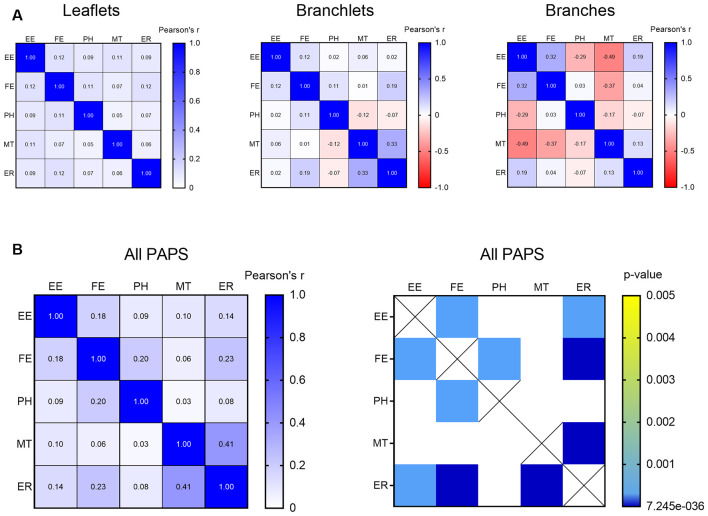
**(A)** Correlation matrix indicating the strength of the correlation between all the described organelles in leaflets, branchlets, and branches separately. **(B)** Correlation matrix indicating strength (left) and significance (right) of the correlation between all the described organelles in all the PAPs pooled together. Only significant correlations after Bonferroni’s correction for multiple comparisons are displayed. Empty endosomes (EE), full endosomes (FE), phagosome (PH), mitochondria (MT), and ER cisternae (ER) for all panels.

In PAPs, organelles can appear alone or be associated with each other within the same PAP. To test the likelihood of finding clusters of multiple organelles and identify which organelles tend to co-occur, we carried out a correlation matrix of the organelle distribution frequency. After correcting for multiple comparisons (Bonferroni), no clusters were found when branches, branchlets, and leaflets were considered independently (Figure 5A). However, when branches, branchlets, and leaflets data were pooled together to increase statistical power we found three main clusters of organelles that significantly co-occurred in PAPs. A first cluster linked together EE, FE, and PH, a second cluster linked EE and FE with ER, and a third cluster linked ER and MT (Figure 5B).

### The Presence of Mitochondrion and Spine Apparatus Influences the Distribution of Some Organelles of the Surrounding PAP

**Figure 6:**
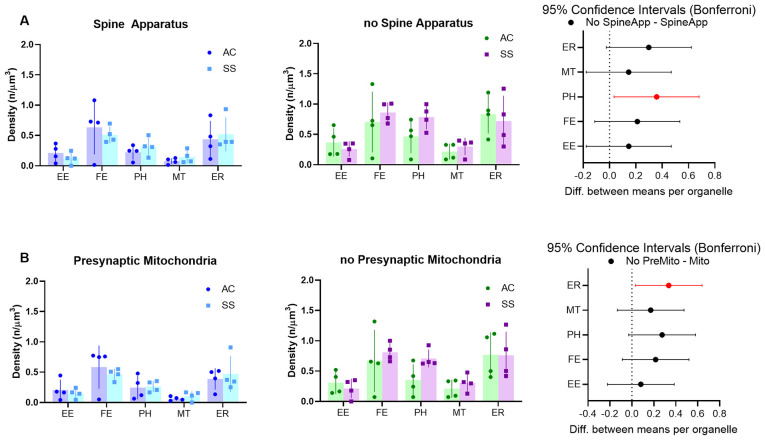
**(A)** Subcellular organelle distribution in PAPs surrounding synapses with (left) or without (middle) spine apparatus and relative statistical analysis (right, rmANOVA followed by *post hoc* Bonferroni tests, *n* = 4). **(B)** Subcellular organelle distribution in PAPs surrounding synapses with (left) or without (middle) presynaptic mitochondrion and relative statistical analysis (right, rmANOVA followed by *post hoc* Bonferroni tests *n* = 4). Empty endosomes (EE), full endosomes (FE), phagosome (PH), mitochondria (MT), and ER cisternae (ER) for all panels.

Astrocytes usually tend to enwrap synapses with their PAPs often reaching the synaptic cleft. Thus, given the strong anatomical and functional interrelationship between neuronal and astrocytic structures, we hypothesized that the PAP’s organelles may play a role in the modulation of synaptic function, and that differences in organelle composition inside the PAP may reflect different needs specific to different types of synapses. To test this hypothesis, we divided synapses based on either the presence of a spine apparatus in the postsynaptic compartment or the presence of a mitochondrion in the presynaptic element and tested whether these different classes of synapses determined a different organelle distribution within the PAPs around them. Given the small number of astrocytic branches surrounding synapses, only branchlets and leaflets were considered for this analysis. Also, their data were pooled together, as their organelle distributions were comparable. Repeated measure three-way ANOVA found a main effect of spine apparatus (*F*_(1,15)_ = 76.62; *p* < 0.0001) and presynaptic mitochondria (*F*_(1,15)_ = 34.30; *p* < 0.0001). *Post hoc* analysis revealed a significant effect for PH (*p* = 0.0226), which occurred more frequently in PAPs contacting synapses without spine apparatus, and for ER, which were more frequent in PAPs contacting synapses without a presynaptic mitochondrion (*p* = 0.0231). None of the other organelles showed a significant difference (Figures 6A,B).

## Discussion

Using volume EM, we described and quantified the distribution of PAPs and their organelles in layer II of the SS and AC cortices. We found that >75% of axo-spinous (putative excitatory) synapses in layer II were contacted by PAPs at the post-synaptic site. Such contacts produced a synaptic coverage that reached the synaptic cleft in about 50% of all synapses and was comparable between the SS and AC cortex. These findings are in line with what was observed previously by our group and others, in different cortices and developmental stages (Genoud et al., 2006; Bernardinelli et al., 2014; Bellesi et al., 2015; Kikuchi et al., 2020). They also indicate that ~50% of cortical synapses are not contacted by PAPs at the level of the synaptic cleft and that ~15% of all synapses are not touched at all by PAPs, thus indicating that the concept of tripartite synapse does not always apply.

Astrocytic processes can be subdivided into branches, branchlets, and leaflets, according to their size and location. In our study, most astrocytic processes contacting the synapses were categorized as leaflets (~60%), ~37% were branchlets and only a minority (<3%) were branches. It is worth mentioning that this classification was based merely on the diameter of these processes (Khakh and Sofroniew, 2015). This distinction was confirmed by measures of surface-to-volumes ratio, whose values were highest in leaflets and lowest in branches, with branchlets being in the middle.

We observed that PAPs contained a variety of organelles such as empty endosomes, full endosomes, phagosomes, mitochondria, and ER cisternae. The number of organelles grew as a function of the process size, with leaflets displaying fewer organelles per unit volume than larger processes. Also, most of the leaflets (~92%) were empty, whereas ~1/3 of the branchlets and about half of the branches had at least one organelle. These results are in line with previous studies showing that most of the subcellular organelles are located in large (>250 nm of diameter) processes (Patrushev et al., 2013). We also observed that the five organelles identified within the PAPs tend to form clusters. Specifically, endosomes appeared often in association with phagosomes, whereas mitochondria were associated with ER cisternae.

These findings challenge previous studies of glial ultrastructure (Peters, 1991), which described PAPs as processes normally lacking organelles like mitochondria, ER, or vesicular structures, although individual vesicles, endosomes, or mitochondria were occasionally mentioned (Peters, 1991; Reichenbach et al., 2010; Bernardinelli et al., 2014; Heller and Rusakov, 2015). Our study suggests differently: if we put together the percentages of leaflets and branchlets, which represent the large majority of PAP contacting synapses, it would seem that about 40% of cortical PAPs contains one or more subcellular organelles. That PAPs are not devoid of organelles has also been reported by other groups. For instance, multiple PAP organelles were recently identified applying novel techniques of tissue fixation, such as high-pressure freezing and freeze substitution that better preserve fine subcellular structure (Zuber et al., 2005; Akagi et al., 2006; Möbius et al., 2010; Sahlender et al., 2014). Similarly, vesicles within the fine astrocytic processes were described in several EM studies performed on “gliosomes,” an *in vitro* preparation of glial subcellular particles often used to study mechanisms of astrocytic transmitter uptake and release (Milanese et al., 2009; Cervetto et al., 2015).

Recent work used array tomography and SBF-SEM to unveil additional morphological features of PAPs which highlighted some new and relevant astrocytic properties (Cahoy et al., 2008; Chung et al., 2013; Davis et al., 2014; Bellesi et al., 2015, 2017). Specifically, it has been discovered that PAPs are capable of structurally remodeling synapses by phagocyting synaptic elements or portions of them (Chung et al., 2013; Bellesi et al., 2017; Lee et al., 2020). This phagocytotic activity requires dedicated intracellular machinery that engulfs, transports, and degrades material within the PAPs. It is not surprising therefore that more than 60% of the organelles that we described within PAPs are related to phagocytosis (EE, FE, and PH). These organelles tend to be associated, thus suggesting that they may be functionally linked and participate in the same biological process (i.e., phagocytosis). Similarly, mitochondria and ER cisternae, which account for about 40% of all the organelles, were often associated within the PAPs. Recent studies have indicated that the interaction between ER cisternae and mitochondria may promote specific astrocytic functions such as brain tissue repair (Göbel et al., 2020). ER cisternae represent the intracellular source of calcium, and calcium activity in astrocytes is largely compartmented and preponderantly occurs in PAPs, while the soma is mostly inactive (Bindocci et al., 2017). Calcium is released intracellularly upon astrocyte activation and concomitant synaptic firing and it is involved in numerous biological processes, including astrocyte metabolic responses and PAPs structural plasticity (Ding et al., 2013; Verkhratsky and Nedergaard, 2018). Therefore, the presence of ER cisternae in PAPs guarantees calcium availability to allow these processes to occur locally. However, since we found evidence of ER cisternae in only ~45% of the PAPs, other mechanisms (e.g., calcium exchangers) must be in place to allow calcium dynamics in ER-free PAPs (Héja and Kardos, 2020).

It is well recognized that PAPs are not only structurally associated with synapses, they fulfill homeostatic, metabolic, and regulatory functions (Kimelberg, 2007; Pellerin et al., 2007; Perea and Araque, 2010). Therefore, the functional state of synapses could influence PAPs functions and vice versa. While it is difficult to identify morphological features that uniquely characterize precise synaptic functional states, some synaptic morphological features define categories of synapses that may behave differently in terms of firing activity or plasticity. This is the case, for instance, for synapses containing a spine apparatus or a presynaptic mitochondrion. The spine apparatus plays a role in synaptic plasticity since synaptopodin-deficient mice that lack these organelles show deficits in long-term potentiation and spatial learning (Jedlicka et al., 2008). Similarly, presynaptic mitochondria are found in axon terminals that are stable and less prone to change in size (Lees et al., 2019). Therefore, synapses characterized by the presence of a spine apparatus or a presynaptic mitochondrion might hold different plastic abilities from those that do not have these organelles. Taking this into account, we analyzed the organelle distribution in PAPs of axo-spinous synapses with or without spine apparatus and presynaptic mitochondria. The distribution was largely similar between these types of synapses, except for phagosomes and ER cisternae (see below). The lack of a large effect can be explained by several reasons: first, PAPs may also be under the influence of nearby synapses that are not necessarily in contact with them. In other words, the distribution of organelles within the PAPs may depend on the overall local environment and not be strictly dependent on the apposed synapses. Second, the presence of presynaptic mitochondria or spine apparatus may induce changes that are too small to be detected in the surrounding PAPs with the current sampling. Third, the organelle distribution is sensitive to synaptic features that we have not considered or are not associated with a specific morphological phenotype (e.g., the amount of neurotransmitter released or the extent of ion changes). The only two changes that we detected were relative to the density of phagosomes and ER cisternae. Phagosomes were more present in synapses lacking the spine apparatus, while ER cisternae were more represented in synapses lacking the presynaptic mitochondrion. We can speculate that both phagosomes and ER in astrocytes could be useful for plastic remodeling of young synapses that still display low levels of pre- and post-synaptic stability.

Growing evidence over the past few years is showing that astrocytes display significant variability in gene expression and physiology within and between brain regions (Miller, 2018). Striking astrocyte heterogeneity has been described among different layers within the same region and different brain regions (hippocampus vs. cerebral cortex; Takata and Hirase, 2008; Tang et al., 2009; John Lin et al., 2017; Clarke et al., 2018). In our study, we sampled PAPs from layer II of the SS and AC cortex, two regions involved in different functions and belonging to a different hierarchical category. We did not observe any difference in PAPs organelle distribution between these cortical regions. Thus, we can speculate that the organelle composition in PAPs is not a heterogeneous feature, at least across the superficial layers of the neocortex.

In conclusion, this study found that an important number of organelles in cortical PAPs are related to phagocytosis, a cellular function that has been only recently described in astrocytes. The organelle distribution in PAPs appears to be not strictly dependent on some specific morphological features of the synapse that the PAPs are enwrapping, leaving open the question of what determines their density and spatial organization. We also acknowledge some limitations of this study. First, SBF-SEM requires a specific EM staining enriched in heavy metals, which gives contrast to lipids facilitating the detection of membranous organelles but also may limit the visualization of protein-enriched structures. This may have caused a reduced detection of synaptic like micro-vesicles of 30–40 nm that have been previously identified in PAPs using other methods (Jourdain et al., 2007; Bergersen et al., 2012; Cervetto et al., 2015). These vesicles are much smaller than our EE (80–150 nm), which probably belong to a completely different category of intracellular PAP organelles with a function that remains to be elucidated. Second, we sampled only PAPs in contact with axo-spinous synapses of layer II, thus the information gathered for the neocortex is incomplete and requires further investigations extending to other cortical layers and types of synapses.

## Data Availability Statement

The raw data supporting the conclusions of this article will be made available by the authors, without undue reservation.

## Ethics Statement

All procedures involving animals adhered to the Animals (Scientific Procedures) Act 1986 and Amendment Regulations 2012 as outlined in UK law and approved by the University of Bristol Animal Welfare and Ethics Review Board.

## Author Contributions

MB, MF, LV, AA, and MJ: conceptualization. AA, MJ, MB, and LV: experiments and analysis. MB, LV, and MF: resources. MB and MF: supervision. MB and LV: writing—original draft. AA, MJ, MF, LV, and MB: writing—review and editing. All authors contributed to the article and approved the submitted version.

## Conflict of Interest

The authors declare that the research was conducted in the absence of any commercial or financial relationships that could be construed as a potential conflict of interest.
